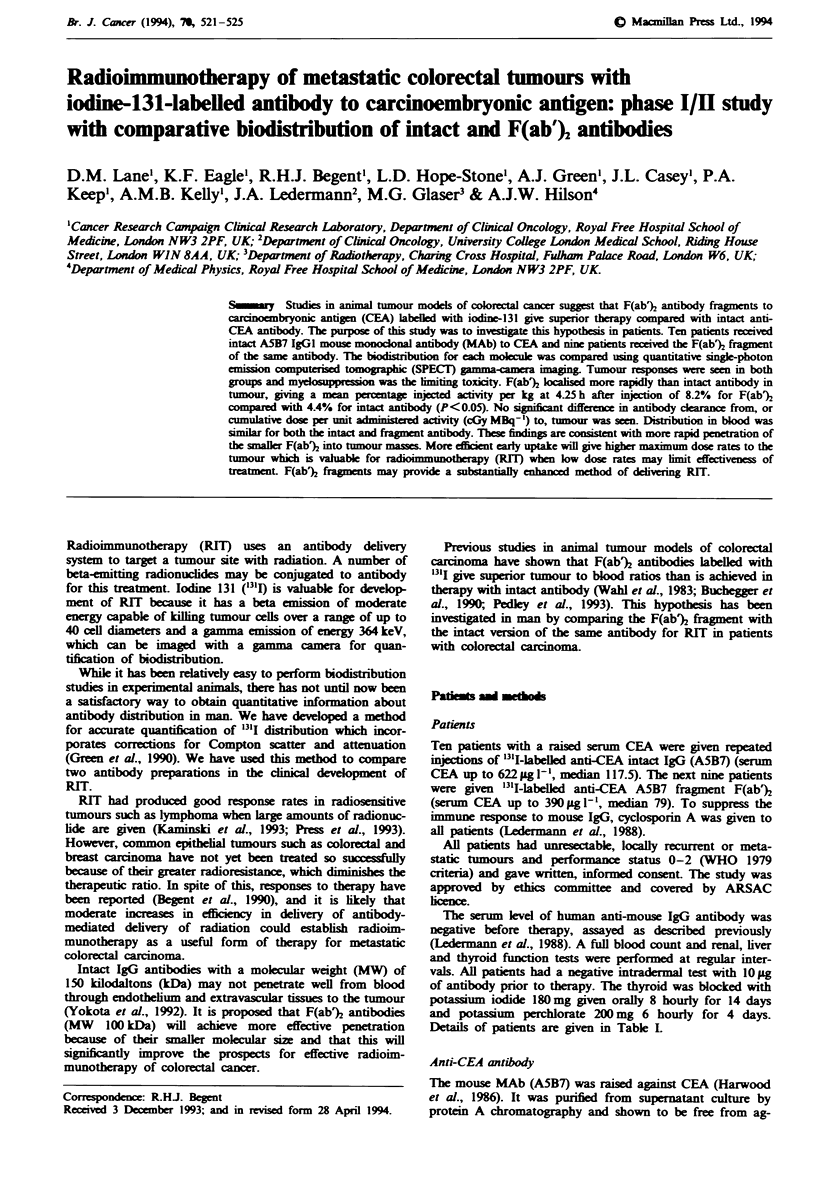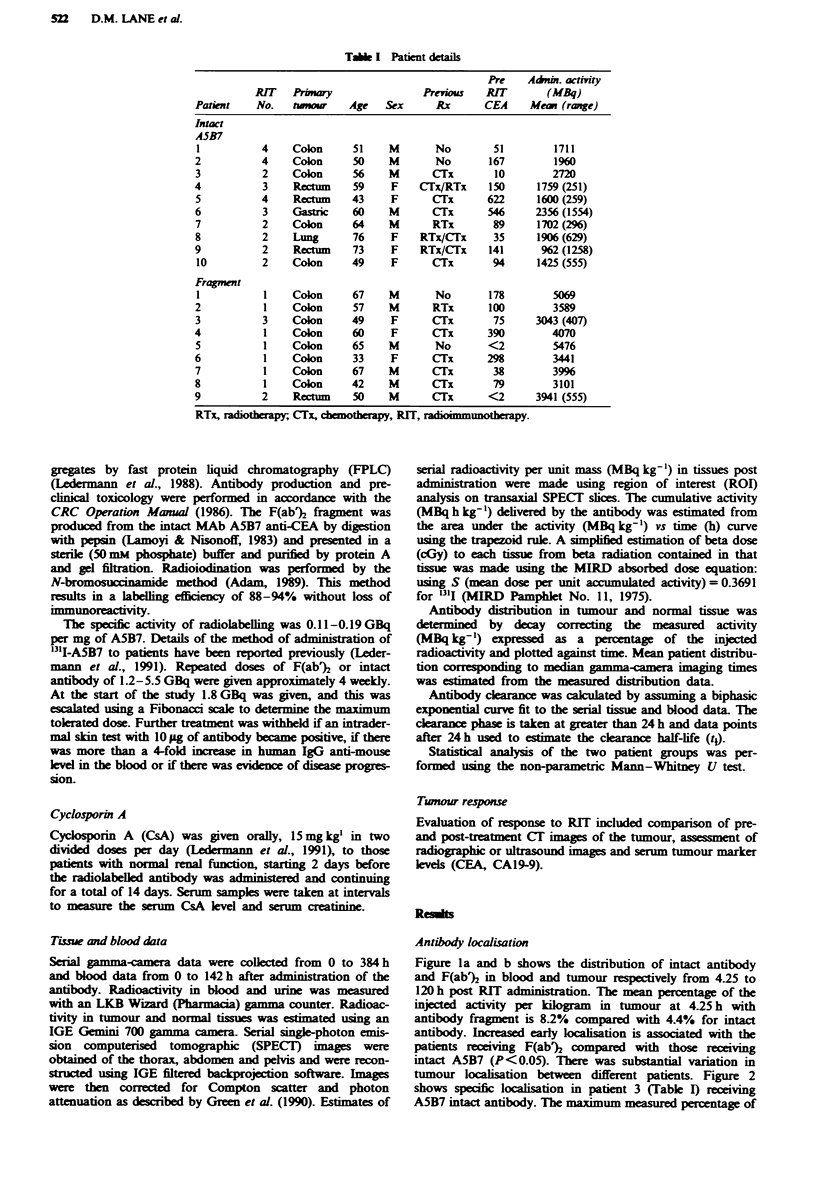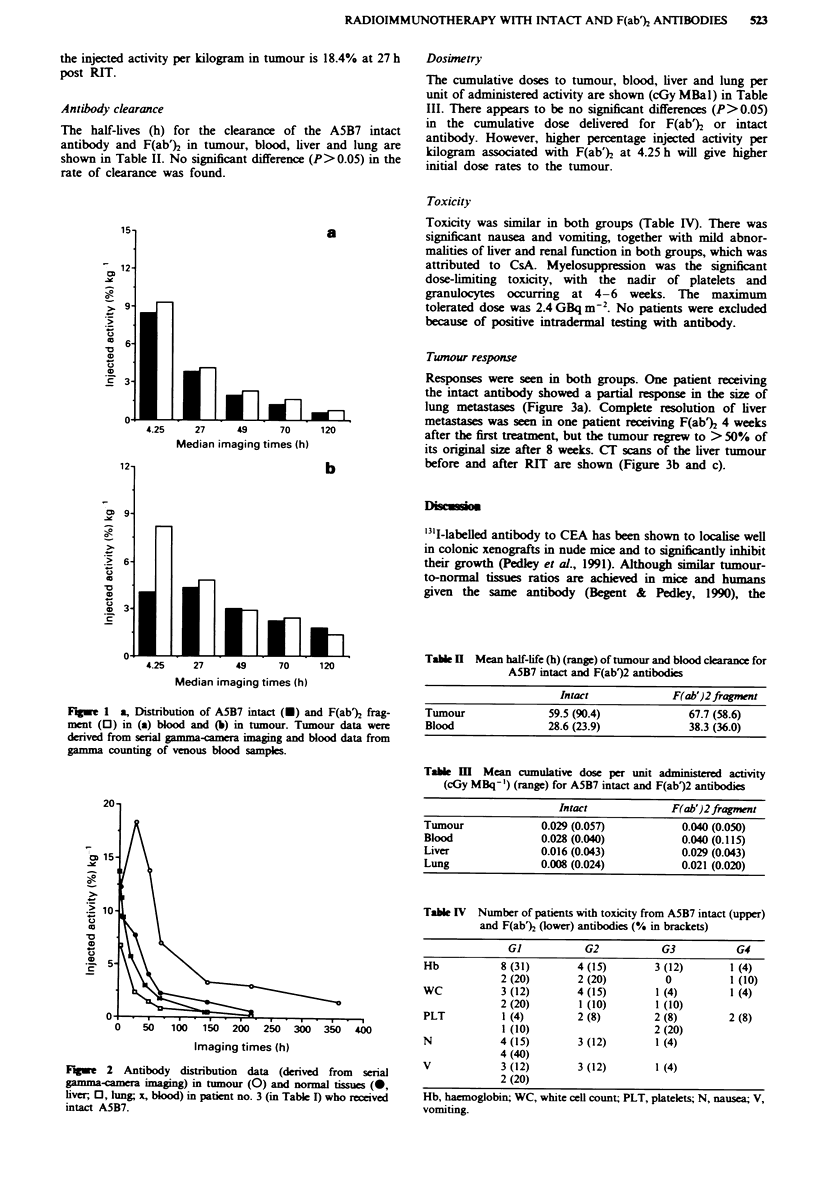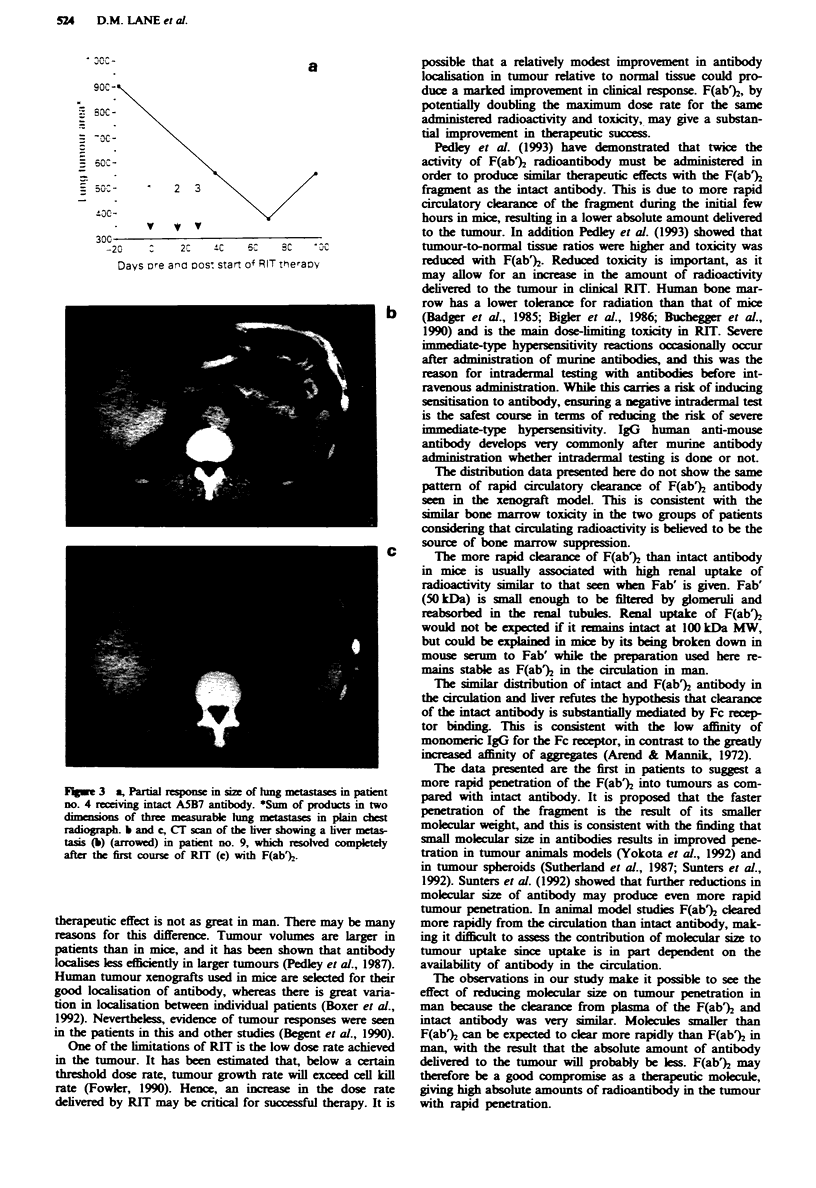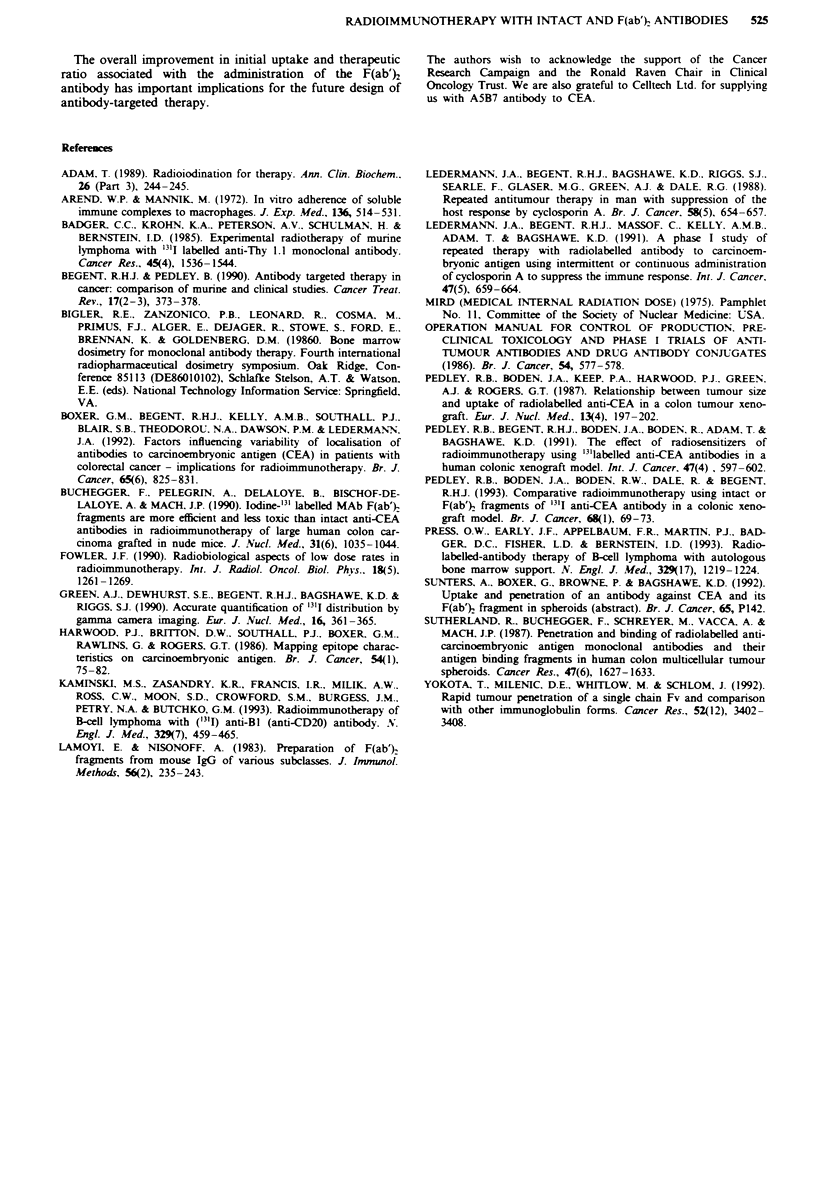# Radioimmunotherapy of metastatic colorectal tumours with iodine-131-labelled antibody to carcinoembryonic antigen: phase I/II study with comparative biodistribution of intact and F(ab')2 antibodies.

**DOI:** 10.1038/bjc.1994.338

**Published:** 1994-09

**Authors:** D. M. Lane, K. F. Eagle, R. H. Begent, L. D. Hope-Stone, A. J. Green, J. L. Casey, P. A. Keep, A. M. Kelly, J. A. Ledermann, M. G. Glaser

**Affiliations:** Department of Clinical Oncology, Royal Free Hospital School of Medicine, London, UK.

## Abstract

**Images:**


Br. J. Cancer (1994), 70, 521-525 © Macmillan Press Ltd., 1994

### Radioimmunotherapy of metastatic colorectal tumours with iodine-131-labelled antibody to carcinoembryonic antigen: phase I/II study with comparative biodistribution of intact and F(ab')$_2$ antibodies

D.M. Lane[1], K.F. Eagle[1], R.H.J. Begent[1], L.D. Hope-Stone[1], A.J. Green[1], J.L. Casey[1], P.A. Keep[1], A.M.B. Kelly[1], J.A. Ledermann[2], M.G. Glaser[3] & A.J.W. Hilson[4]

[1]*Cancer Research Campaign Clinical Research Laboratory, Department of Clinical Oncology, Royal Free Hospital School of Medicine, London NW3 2PF, UK;* [2]*Department of Clinical Oncology, University College London Medical School, Riding House Street, London W1N 8AA, UK;* [3]*Department of Radiotherapy, Charing Cross Hospital, Fulham Palace Road, London W6, UK;* [4]*Department of Medical Physics, Royal Free Hospital School of Medicine, London NW3 2PF, UK.*

**Summary** Studies in animal tumour models of colorectal cancer suggest that F(ab')$_2$ antibody fragments to carcinoembryonic antigen (CEA) labelled with iodine-131 give superior therapy compared with intact anti-CEA antibody. The purpose of this study was to investigate this hypothesis in patients. Ten patients received intact A5B7 IgG1 mouse monoclonal antibody (MAb) to CEA and nine patients received the F(ab')$_2$ fragment of the same antibody. The biodistribution for each molecule was compared using quantitative single-photon emission computerised tomographic (SPECT) gamma-camera imaging. Tumour responses were seen in both groups and myelosuppression was the limiting toxicity. F(ab')$_2$ localised more rapidly than intact antibody in tumour, giving a mean percentage injected activity per kg at 4.25 h after injection of 8.2% for F(ab')$_2$ compared with 4.4% for intact antibody ($P<0.05$). No significant difference in antibody clearance from, or cumulative dose per unit administered activity (cGy MBq$^{-1}$) to, tumour was seen. Distribution in blood was similar for both the intact and fragment antibody. These findings are consistent with more rapid penetration of the smaller F(ab')$_2$ into tumour masses. More efficient early uptake will give higher maximum dose rates to the tumour which is valuable for radioimmunotherapy (RIT) when low dose rates may limit effectiveness of treatment. F(ab')$_2$ fragments may provide a substantially enhanced method of delivering RIT.

Radioimmunotherapy (RIT) uses an antibody delivery system to target a tumour site with radiation. A number of beta-emitting radionuclides may be conjugated to antibody for this treatment. Iodine 131 ($^{131}$I) is valuable for development of RIT because it has a beta emission of moderate energy capable of killing tumour cells over a range of up to 40 cell diameters and a gamma emission of energy 364 keV, which can be imaged with a gamma camera for quantification of biodistribution.

While it has been relatively easy to perform biodistribution studies in experimental animals, there has not until now been a satisfactory way to obtain quantitative information about antibody distribution in man. We have developed a method for accurate quantification of $^{131}$I distribution which incorporates corrections for Compton scatter and attenuation (Green *et al.*, 1990). We have used this method to compare two antibody preparations in the clinical development of RIT.

RIT had produced good response rates in radiosensitive tumours such as lymphoma when large amounts of radionuclide are given (Kaminski *et al.*, 1993; Press *et al.*, 1993). However, common epithelial tumours such as colorectal and breast carcinoma have not yet been treated so successfully because of their greater radioresistance, which diminishes the therapeutic ratio. In spite of this, responses to therapy have been reported (Begent *et al.*, 1990), and it is likely that moderate increases in efficiency in delivery of antibody-mediated delivery of radiation could establish radioimmunotherapy as a useful form of therapy for metastatic colorectal carcinoma.

Intact IgG antibodies with a molecular weight (MW) of 150 kilodaltons (kDa) may not penetrate well from blood through endothelium and extravascular tissues to the tumour (Yokota *et al.*, 1992). It is proposed that F(ab')$_2$ antibodies (MW 100 kDa) will achieve more effective penetration because of their smaller molecular size and that this will significantly improve the prospects for effective radioimmunotherapy of colorectal cancer.

Correspondence: R.H.J. Begent

Received 3 December 1993; and in revised form 28 April 1994.

Previous studies in animal tumour models of colorectal carcinoma have shown that F(ab')$_2$ antibodies labelled with $^{131}$I give superior tumour to blood ratios than is achieved in therapy with intact antibody (Wahl *et al.*, 1983; Buchegger *et al.*, 1990; Pedley *et al.*, 1993). This hypothesis has been investigated in man by comparing the F(ab')$_2$ fragment with the intact version of the same antibody for RIT in patients with colorectal carcinoma.

### Patients and methods

### *Patients*

Ten patients with a raised serum CEA were given repeated injections of $^{131}$I-labelled anti-CEA intact IgG (A5B7) (serum CEA up to 622 $\mu$g l$^{-1}$, median 117.5). The next nine patients were given $^{131}$I-labelled anti-CEA A5B7 fragment F(ab')$_2$ (serum CEA up to 390 $\mu$g l$^{-1}$, median 79). To suppress the immune response to mouse IgG, cyclosporin A was given to all patients (Ledermann *et al.*, 1988).

All patients had unresectable, locally recurrent or metastatic tumours and performance status 0-2 (WHO 1979 criteria) and gave written, informed consent. The study was approved by ethics committee and covered by ARSAC licence.

The serum level of human anti-mouse IgG antibody was negative before therapy, assayed as described previously (Ledermann *et al.*, 1988). A full blood count and renal, liver and thyroid function tests were performed at regular intervals. All patients had a negative intradermal test with 10 $\mu$g of antibody prior to therapy. The thyroid was blocked with potassium iodide 180 mg given orally 8 hourly for 14 days and potassium perchlorate 200 mg 6 hourly for 4 days. Details of patients are given in Table I.

### *Anti-CEA antibody*

The mouse MAb (A5B7) was raised against CEA (Harwood *et al.*, 1986). It was purified from supernatant culture by protein A chromatography and shown to be free from ag-

**Table I** Patient details

| Patient | RIT No. | Primary tumour | Age | Sex | Previous Rx | Pre RIT CEA | Admin. activity (MBq) Mean (range) |
|---|---|---|---|---|---|---|---|
| *Intact A5B7* | | | | | | | |
| 1 | 4 | Colon | 51 | M | No | 51 | 1711 |
| 2 | 4 | Colon | 50 | M | No | 167 | 1960 |
| 3 | 2 | Colon | 56 | M | CTx | 10 | 2720 |
| 4 | 3 | Rectum | 59 | F | CTx/RTx | 150 | 1759 (251) |
| 5 | 4 | Rectum | 43 | F | CTx | 622 | 1600 (259) |
| 6 | 3 | Gastric | 60 | M | CTx | 546 | 2356 (1554) |
| 7 | 2 | Colon | 64 | M | RTx | 89 | 1702 (296) |
| 8 | 2 | Lung | 76 | F | RTx/CTx | 35 | 1906 (629) |
| 9 | 2 | Rectum | 73 | F | RTx/CTx | 141 | 962 (1258) |
| 10 | 2 | Colon | 49 | F | CTx | 94 | 1425 (555) |
| *Fragment* | | | | | | | |
| 1 | 1 | Colon | 67 | M | No | 178 | 5069 |
| 2 | 1 | Colon | 57 | M | RTx | 100 | 3589 |
| 3 | 3 | Colon | 49 | F | CTx | 75 | 3043 (407) |
| 4 | 1 | Colon | 60 | F | CTx | 390 | 4070 |
| 5 | 1 | Colon | 65 | M | No | <2 | 5476 |
| 6 | 1 | Colon | 33 | F | CTx | 298 | 3441 |
| 7 | 1 | Colon | 67 | M | CTx | 38 | 3996 |
| 8 | 1 | Colon | 42 | M | CTx | 79 | 3101 |
| 9 | 2 | Rectum | 50 | M | CTx | <2 | 3941 (555) |

RTx, radiotherapy; CTx, chemotherapy, RIT, radioimmunotherapy.

gregates by fast protein liquid chromatography (FPLC) (Ledermann *et al.*, 1988). Antibody production and pre-clinical toxicology were performed in accordance with the *CRC Operation Manual* (1986). The $F(ab')_2$ fragment was produced from the intact MAb A5B7 anti-CEA by digestion with pepsin (Lamoyi & Nisonoff, 1983) and presented in a sterile (50 mM phosphate) buffer and purified by protein A and gel filtration. Radioiodination was performed by the *N*-bromosuccinamide method (Adam, 1989). This method results in a labelling efficiency of 88–94% without loss of immunoreactivity.

The specific activity of radiolabelling was 0.11–0.19 GBq per mg of A5B7. Details of the method of administration of $^{131}$I-A5B7 to patients have been reported previously (Ledermann *et al.*, 1991). Repeated doses of $F(ab')_2$ or intact antibody of 1.2–5.5 GBq were given approximately 4 weekly. At the start of the study 1.8 GBq was given, and this was escalated using a Fibonacci scale to determine the maximum tolerated dose. Further treatment was withheld if an intradermal skin test with 10 μg of antibody became positive, if there was more than a 4-fold increase in human IgG anti-mouse level in the blood or if there was evidence of disease progression.

### *Cyclosporin A*

Cyclosporin A (CsA) was given orally, 15 mg kg$^{1}$ in two divided doses per day (Ledermann *et al.*, 1991), to those patients with normal renal function, starting 2 days before the radiolabelled antibody was administered and continuing for a total of 14 days. Serum samples were taken at intervals to measure the serum CsA level and serum creatinine.

### *Tissue and blood data*

Serial gamma-camera data were collected from 0 to 384 h and blood data from 0 to 142 h after administration of the antibody. Radioactivity in blood and urine was measured with an LKB Wizard (Pharmacia) gamma counter. Radioactivity in tumour and normal tissues was estimated using an IGE Gemini 700 gamma camera. Serial single-photon emission computerised tomographic (SPECT) images were obtained of the thorax, abdomen and pelvis and were reconstructed using IGE filtered backprojection software. Images were then corrected for Compton scatter and photon attenuation as described by Green *et al.* (1990). Estimates of serial radioactivity per unit mass (MBq $kg^{-1}$) in tissues post administration were made using region of interest (ROI) analysis on transaxial SPECT slices. The cumulative activity (MBq h $kg^{-1}$) delivered by the antibody was estimated from the area under the activity (MBq $kg^{-1}$) *vs* time (h) curve using the trapezoid rule. A simplified estimation of beta dose (cGy) to each tissue from beta radiation contained in that tissue was made using the MIRD absorbed dose equation: using $S$ (mean dose per unit accumulated activity) = 0.3691 for $^{131}$I (MIRD Pamphlet No. 11, 1975).

Antibody distribution in tumour and normal tissue was determined by decay correcting the measured activity (MBq $kg^{-1}$) expressed as a percentage of the injected radioactivity and plotted against time. Mean patient distribution corresponding to median gamma-camera imaging times was estimated from the measured distribution data.

Antibody clearance was calculated by assuming a biphasic exponential curve fit to the serial tissue and blood data. The clearance phase is taken at greater than 24 h and data points after 24 h used to estimate the clearance half-life ($t_{½}$).

Statistical analysis of the two patient groups was performed using the non-parametric Mann–Whitney $U$ test.

### *Tumour response*

Evaluation of response to RIT included comparison of pre- and post-treatment CT images of the tumour, assessment of radiographic or ultrasound images and serum tumour marker levels (CEA, CA19-9).

### Results

### *Antibody localisation*

Figure 1a and b shows the distribution of intact antibody and $F(ab')_2$ in blood and tumour respectively from 4.25 to 120 h post RIT administration. The mean percentage of the injected activity per kilogram in tumour at 4.25 h with antibody fragment is 8.2% compared with 4.4% for intact antibody. Increased early localisation is associated with the patients receiving $F(ab')_2$ compared with those receiving intact A5B7 ($P < 0.05$). There was substantial variation in tumour localisation between different patients. Figure 2 shows specific localisation in patient 3 (Table I) receiving A5B7 intact antibody. The maximum measured percentage of the injected activity per kilogram in tumour is 18.4% at 27 h post RIT.

*Antibody clearance*

The half-lives (h) for the clearance of the A5B7 intact antibody and F(ab')$_2$ in tumour, blood, liver and lung are shown in Table II. No significant difference ($P > 0.05$) in the rate of clearance was found.

**Figure 1** a, Distribution of A5B7 intact (■) and F(ab')$_2$ fragment (□) in (a) blood and (b) in tumour. Tumour data were derived from serial gamma-camera imaging and blood data from gamma counting of venous blood samples.

**Figure 2** Antibody distribution data (derived from serial gamma-camera imaging) in tumour (○) and normal tissues (●, liver; □, lung; x, blood) in patient no. 3 (in Table I) who received intact A5B7.

*Dosimetry*

The cumulative doses to tumour, blood, liver and lung per unit of administered activity are shown (cGy MBa1) in Table III. There appears to be no significant differences ($P > 0.05$) in the cumulative dose delivered for F(ab')$_2$ or intact antibody. However, higher percentage injected activity per kilogram associated with F(ab')$_2$ at 4.25 h will give higher initial dose rates to the tumour.

*Toxicity*

Toxicity was similar in both groups (Table IV). There was significant nausea and vomiting, together with mild abnormalities of liver and renal function in both groups, which was attributed to CsA. Myelosuppression was the significant dose-limiting toxicity, with the nadir of platelets and granulocytes occurring at 4–6 weeks. The maximum tolerated dose was 2.4 GBq m$^{-2}$. No patients were excluded because of positive intradermal testing with antibody.

*Tumour response*

Responses were seen in both groups. One patient receiving the intact antibody showed a partial response in the size of lung metastases (Figure 3a). Complete resolution of liver metastases was seen in one patient receiving F(ab')$_2$ 4 weeks after the first treatment, but the tumour regrew to $> 50\%$ of its original size after 8 weeks. CT scans of the liver tumour before and after RIT are shown (Figure 3b and c).

### Discussion

$^{131}$I-labelled antibody to CEA has been shown to localise well in colonic xenografts in nude mice and to significantly inhibit their growth (Pedley *et al.*, 1991). Although similar tumour-to-normal tissues ratios are achieved in mice and humans given the same antibody (Begent & Pedley, 1990), the

**Table II** Mean half-life (h) (range) of tumour and blood clearance for A5B7 intact and F(ab')2 antibodies

| | *Intact* | *F(ab')2 fragment* |
|---|---|---|
| Tumour | 59.5 (90.4) | 67.7 (58.6) |
| Blood | 28.6 (23.9) | 38.3 (36.0) |

**Table III** Mean cumulative dose per unit administered activity (cGy MBq$^{-1}$) (range) for A5B7 intact and F(ab')2 antibodies

| | *Intact* | *F(ab')2 fragment* |
|---|---|---|
| Tumour | 0.029 (0.057) | 0.040 (0.050) |
| Blood | 0.028 (0.040) | 0.040 (0.115) |
| Liver | 0.016 (0.043) | 0.029 (0.043) |
| Lung | 0.008 (0.024) | 0.021 (0.020) |

**Table IV** Number of patients with toxicity from A5B7 intact (upper) and F(ab')$_2$ (lower) antibodies (% in brackets)

| | *G1* | *G2* | *G3* | *G4* |
|---|---|---|---|---|
| Hb | 8 (31) | 4 (15) | 3 (12) | 1 (4) |
| | 2 (20) | 2 (20) | 0 | 1 (10) |
| WC | 3 (12) | 4 (15) | 1 (4) | 1 (4) |
| | 2 (20) | 1 (10) | 1 (10) | |
| PLT | 1 (4) | 2 (8) | 2 (8) | 2 (8) |
| | 1 (10) | | 2 (20) | |
| N | 4 (15) | 3 (12) | 1 (4) | |
| | 4 (40) | | | |
| V | 3 (12) | 3 (12) | 1 (4) | |
| | 2 (20) | | | |

Hb, haemoglobin; WC, white cell count; PLT, platelets; N, nausea; V, vomiting.

**Figure 3** a, Partial response in size of lung metastases in patient no. 4 receiving intact A5B7 antibody. *Sum of products in two dimensions of three measurable lung metastases in plain chest radiograph. b and c, CT scan of the liver showing a liver metastasis (b) (arrowed) in patient no. 9, which resolved completely after the first course of RIT (c) with F(ab')$_2$.

therapeutic effect is not as great in man. There may be many reasons for this difference. Tumour volumes are larger in patients than in mice, and it has been shown that antibody localises less efficiently in larger tumours (Pedley *et al.*, 1987). Human tumour xenografts used in mice are selected for their good localisation of antibody, whereas there is great variation in localisation between individual patients (Boxer *et al.*, 1992). Nevertheless, evidence of tumour responses were seen in the patients in this and other studies (Begent *et al.*, 1990).

One of the limitations of RIT is the low dose rate achieved in the tumour. It has been estimated that, below a certain threshold dose rate, tumour growth rate will exceed cell kill rate (Fowler, 1990). Hence, an increase in the dose rate delivered by RIT may be critical for successful therapy. It is possible that a relatively modest improvement in antibody localisation in tumour relative to normal tissue could produce a marked improvement in clinical response. F(ab')$_2$, by potentially doubling the maximum dose rate for the same administered radioactivity and toxicity, may give a substantial improvement in therapeutic success.

Pedley *et al.* (1993) have demonstrated that twice the activity of F(ab')$_2$ radioantibody must be administered in order to produce similar therapeutic effects with the F(ab')$_2$ fragment as the intact antibody. This is due to more rapid circulatory clearance of the fragment during the initial few hours in mice, resulting in a lower absolute amount delivered to the tumour. In addition Pedley *et al.* (1993) showed that tumour-to-normal tissue ratios were higher and toxicity was reduced with F(ab')$_2$. Reduced toxicity is important, as it may allow for an increase in the amount of radioactivity delivered to the tumour in clinical RIT. Human bone marrow has a lower tolerance for radiation than that of mice (Badger *et al.*, 1985; Bigler *et al.*, 1986; Buchegger *et al.*, 1990) and is the main dose-limiting toxicity in RIT. Severe immediate-type hypersensitivity reactions occasionally occur after administration of murine antibodies, and this was the reason for intradermal testing with antibodies before intravenous administration. While this carries a risk of inducing sensitisation to antibody, ensuring a negative intradermal test is the safest course in terms of reducing the risk of severe immediate-type hypersensitivity. IgG human anti-mouse antibody develops very commonly after murine antibody administration whether intradermal testing is done or not.

The distribution data presented here do not show the same pattern of rapid circulatory clearance of F(ab')$_2$ antibody seen in the xenograft model. This is consistent with the similar bone marrow toxicity in the two groups of patients considering that circulating radioactivity is believed to be the source of bone marrow suppression.

The more rapid clearance of F(ab')$_2$ than intact antibody in mice is usually associated with high renal uptake of radioactivity similar to that seen when Fab' is given. Fab' (50 kDa) is small enough to be filtered by glomeruli and reabsorbed in the renal tubules. Renal uptake of F(ab')$_2$ would not be expected if it remains intact at 100 kDa MW, but could be explained in mice by its being broken down in mouse serum to Fab' while the preparation used here remains stable as F(ab')$_2$ in the circulation in man.

The similar distribution of intact and F(ab')$_2$ antibody in the circulation and liver refutes the hypothesis that clearance of the intact antibody is substantially mediated by Fc receptor binding. This is consistent with the low affinity of monomeric IgG for the Fc receptor, in contrast to the greatly increased affinity of aggregates (Arend & Mannik, 1972).

The data presented are the first in patients to suggest a more rapid penetration of the F(ab')$_2$ into tumours as compared with intact antibody. It is proposed that the faster penetration of the fragment is the result of its smaller molecular weight, and this is consistent with the finding that small molecular size in antibodies results in improved penetration in tumour animals models (Yokota *et al.*, 1992) and in tumour spheroids (Sutherland *et al.*, 1987; Sunters *et al.*, 1992). Sunters *et al.* (1992) showed that further reductions in molecular size of antibody may produce even more rapid tumour penetration. In animal model studies F(ab')$_2$ cleared more rapidly from the circulation than intact antibody, making it difficult to assess the contribution of molecular size to tumour uptake since uptake is in part dependent on the availability of antibody in the circulation.

The observations in our study make it possible to see the effect of reducing molecular size on tumour penetration in man because the clearance from plasma of the F(ab')$_2$ and intact antibody was very similar. Molecules smaller than F(ab')$_2$ can be expected to clear more rapidly than F(ab')$_2$ in man, with the result that the absolute amount of antibody delivered to the tumour will probably be less. F(ab')$_2$ may therefore be a good compromise as a therapeutic molecule, giving high absolute amounts of radioantibody in the tumour with rapid penetration.

The overall improvement in initial uptake and therapeutic ratio associated with the administration of the F(ab')$_2$ antibody has important implications for the future design of antibody-targeted therapy.

The authors wish to acknowledge the support of the Cancer Research Campaign and the Ronald Raven Chair in Clinical Oncology Trust. We are also grateful to Celltech Ltd. for supplying us with A5B7 antibody to CEA.

### References

ADAM, T. (1989). Radioiodination for therapy. *Ann. Clin. Biochem.*, **26** (Part 3), 244–245.

AREND, W.P. & MANNIK, M. (1972). In vitro adherence of soluble immune complexes to macrophages. *J. Exp. Med.*, **136**, 514–531.

BADGER, C.C., KROHN, K.A., PETERSON, A.V., SCHULMAN, H. & BERNSTEIN, I.D. (1985). Experimental radiotherapy of murine lymphoma with $^{131}$I labelled anti-Thy 1.1 monoclonal antibody. *Cancer Res.*, **45**(4), 1536–1544.

BEGENT, R.H.J. & PEDLEY, B. (1990). Antibody targeted therapy in cancer: comparison of murine and clinical studies. *Cancer Treat. Rev.*, **17**(2–3), 373–378.

BIGLER, R.E., ZANZONICO, P.B., LEONARD, R., COSMA, M., PRIMUS, F.J., ALGER, E., DEJAGER, R., STOWE, S., FORD, E., BRENNAN, K. & GOLDENBERG, D.M. (19860. Bone marrow dosimetry for monoclonal antibody therapy. Fourth international radiopharmaceutical dosimetry symposium. Oak Ridge, Conference 85113 (DE86010102), Schlafke Stelson, A.T. & Watson, E.E. (eds). National Technology Information Service: Springfield, VA.

BOXER, G.M., BEGENT, R.H.J., KELLY, A.M.B., SOUTHALL, P.J., BLAIR, S.B., THEODOROU, N.A., DAWSON, P.M. & LEDERMANN, J.A. (1992). Factors influencing variability of localisation of antibodies to carcinoembryonic antigen (CEA) in patients with colorectal cancer – implications for radioimmunotherapy. *Br. J. Cancer*, **65**(6), 825–831.

BUCHEGGER, F., PELEGRIN, A., DELALOYE, B., BISCHOF-DELALOYE, A. & MACH, J.P. (1990). Iodine-$^{131}$ labelled MAb F(ab')$_2$ fragments are more efficient and less toxic than intact anti-CEA antibodies in radioimmunotherapy of large human colon carcinoma grafted in nude mice. *J. Nucl. Med.*, **31**(6), 1035–1044.

FOWLER, J.F. (1990). Radiobiological aspects of low dose rates in radioimmunotherapy. *Int. J. Radiol. Oncol. Biol. Phys.*, **18**(5), 1261–1269.

GREEN, A.J., DEWHURST, S.E., BEGENT, R.H.J., BAGSHAWE, K.D. & RIGGS, S.J. (1990). Accurate quantification of $^{131}$I distribution by gamma camera imaging. *Eur. J. Nucl. Med.*, **16**, 361–365.

HARWOOD, P.J., BRITTON, D.W., SOUTHALL, P.J., BOXER, G.M., RAWLINS, G. & ROGERS, G.T. (1986). Mapping epitope characteristics on carcinoembryonic antigen. *Br. J. Cancer*, **54**(1), 75–82.

KAMINSKI, M.S., ZASANDRY, K.R., FRANCIS, I.R., MILIK, A.W., ROSS, C.W., MOON, S.D., CROWFORD, S.M., BURGESS, J.M., PETRY, N.A. & BUTCHKO, G.M. (1993). Radioimmunotherapy of B-cell lymphoma with ($^{131}$I) anti-B1 (anti-CD20) antibody. *N. Engl. J. Med.*, **329**(7), 459–465.

LAMOYI, E. & NISONOFF, A. (1983). Preparation of F(ab')$_2$ fragments from mouse IgG of various subclasses. *J. Immunol. Methods*, **56**(2), 235–243.

LEDERMANN, J.A., BEGENT, R.H.J., BAGSHAWE, K.D., RIGGS, S.J., SEARLE, F., GLASER, M.G., GREEN, A.J. & DALE, R.G. (1988). Repeated antitumour therapy in man with suppression of the host response by cyclosporin A. *Br. J. Cancer*, **58**(5), 654–657.

LEDERMANN, J.A., BEGENT, R.H.J., MASSOF, C., KELLY, A.M.B., ADAM, T. & BAGSHAWE, K.D. (1991). A phase I study of repeated therapy with radiolabelled antibody to carcinoembryonic antigen using intermittent or continuous administration of cyclosporin A to suppress the immune response. *Int. J. Cancer*, **47**(5), 659–664.

MIRD (MEDICAL INTERNAL RADIATION DOSE) (1975). Pamphlet No. 11, Committee of the Society of Nuclear Medicine: USA.

OPERATION MANUAL FOR CONTROL OF PRODUCTION, PRECLINICAL TOXICOLOGY AND PHASE I TRIALS OF ANTI-TUMOUR ANTIBODIES AND DRUG ANTIBODY CONJUGATES (1986). *Br. J. Cancer*, **54**, 577–578.

PEDLEY, R.B., BODEN, J.A., KEEP, P.A., HARWOOD, P.J., GREEN, A.J. & ROGERS, G.T. (1987). Relationship between tumour size and uptake of radiolabelled anti-CEA in a colon tumour xenograft. *Eur. J. Nucl. Med.*, **13**(4), 197–202.

PEDLEY, R.B., BEGENT, R.H.J., BODEN, J.A., BODEN, R., ADAM, T. & BAGSHAWE, K.D. (1991). The effect of radiosensitizers of radioimmunotherapy using $^{131}$labelled anti-CEA antibodies in a human colonic xenograft model. *Int. J. Cancer*, **47**(4) , 597–602.

PEDLEY, R.B., BODEN, J.A., BODEN, R.W., DALE, R. & BEGENT, R.H.J. (1993). Comparative radioimmunotherapy using intact or F(ab')$_2$ fragments of $^{131}$I anti-CEA antibody in a colonic xenograft model. *Br. J. Cancer*, **68**(1), 69–73.

PRESS, O.W., EARLY, J.F., APPELBAUM, F.R., MARTIN, P.J., BADGER, D.C., FISHER, L.D. & BERNSTEIN, I.D. (1993). Radiolabelled-antibody therapy of B-cell lymphoma with autologous bone marrow support. *N. Engl. J. Med.*, **329**(17), 1219–1224.

SUNTERS, A., BOXER, G., BROWNE, P. & BAGSHAWE, K.D. (1992). Uptake and penetration of an antibody against CEA and its F(ab')$_2$ fragment in spheroids (abstract). *Br. J. Cancer*, **65**, P142.

SUTHERLAND, R., BUCHEGGER, F., SCHREYER, M., VACCA, A. & MACH, J.P. (1987). Penetration and binding of radiolabelled anti-carcinoembryonic antigen monoclonal antibodies and their antigen binding fragments in human colon multicellular tumour spheroids. *Cancer Res.*, **47**(6), 1627–1633.

YOKOTA, T., MILENIC, D.E., WHITLOW, M. & SCHLOM, J. (1992). Rapid tumour penetration of a single chain Fv and comparison with other immunoglobulin forms. *Cancer Res.*, **52**(12), 3402–3408.